# Key operational characteristics in emergency department observation units: a comparative study between sites in the United States and Asia

**DOI:** 10.1186/1865-1380-7-6

**Published:** 2014-02-05

**Authors:** Atthasit Komindr, Christopher W Baugh, Shamai A Grossman, J Stephen Bohan

**Affiliations:** 1 Emergency Unit, King Chulalongkorn Memorial Hospital, 1873 Rama 4 Road, Pathumwan, Bangkok 10330, Thailand; 2 Department of Emergency Medicine, Brigham and Women’s Hospital, 75 Francis Street, Boston, MA 02115, USA; 3 Department of Emergency Medicine, Beth Israel Deaconess Medical Center, one deaconess road WCC2, Boston, MA 02215, USA

**Keywords:** Emergency department, Management, Metrics, Observation units, Operations

## Abstract

**Background:**

To improve efficiency, emergency departments (EDs) use dedicated observation units (OUs) to manage patients who are unable to be discharged home, yet do not clearly require inpatient hospitalization. However, operational metrics and their ideal targets have not been created for this setting and patient population. Variation in these metrics across different countries has not previously been reported. This study aims to define and compare key operational characteristics between three ED OUs in the United States (US) and three ED OUs in Asia.

**Methods:**

This is a descriptive study of six tertiary-care hospitals, all of which are level 1 trauma centers and have OUs managed by ED staff. We collected data via various methods, including a standardized survey, direct observation, and interviews with unit leadership, and compared these data across continents.

**Results:**

We define multiple key operational characteristics to compare between sites, including OU length of stay (LOS), OU discharge rate, and bed turnover rate. OU LOS in the US and Asian sites averaged 12.9 hours (95% CI, 8.3 to 17.5) and 20.5 hours (95% CI, -49.4 to 90.4), respectively (*P* = 0.39). OU discharge rates in the US and Asia averaged 84.3% (95% CI, 81.5 to 87.2) and 88.7% (95% CI, 81.5 to 95.8), respectively (*P* = 0.11), and the bed turnover rates in the US and Asian sites averaged 1.6 patients/bed/day (95% CI, -0.1 to 3.3) and 0.9 patient/bed/day (95% CI, -0.6 to 2.4), respectively (*P* = 0.27).

**Conclusions:**

Prior research has shown that the OU is a resource that can mitigate many of problems in the ED and hospital, while simultaneously improving patient care and satisfaction. We describe key operational characteristics that are relevant to all OUs, regardless of geography or healthcare system to monitor and maximize efficiency. Although measures of LOS and bed turnover varied widely between US and Asian sites, we did not find a statistically significant difference. Use of these metrics may enable hospitals to establish or revise an ED OU and reduce OU LOS, increase bed turnover, and discharge rates while simultaneously improving patient satisfaction and quality of care.

## Background

Providers on different continents and in varied countries manage health care systems and hospital operations in unique ways despite sharing many common problems. Emergency department (ED) patient volume has continued to grow yearly worldwide [1]. This trend has caused many of the same challenges, namely ED overcrowding, compromised patient care, decreased patient satisfaction, and shortages of ED and hospital personnel, regardless of location [2,3]. Although many of these issues lack an obvious or feasible solution, the observation unit (OU) has been shown to be among the most promising tools to address many of these problems [4-8].

A dedicated OU is an area in the hospital, usually located within or adjacent to the ED, where patients can receive additional therapies, clinical observation, and diagnostics following an ED visit. Most OU’s are managed by the ED [9,10]. Typically, patients stay for up to 24 hours and are safely discharged home, avoiding the need for an inpatient admission [11]. Common conditions managed and observed in an OU include chest pain, abdominal pain, asthma, head injury, and transient ischemic attacks [11]. Numerous studies have shown that many other conditions and complaints can also be effectively and efficiently managed in this setting [7,12-14]. The average cost savings of an OU stay is nearly $1,600 USD compared to an inpatient hospitalization [15,16]. Additionally, studies have shown equivalent clinical outcomes and even higher patient satisfaction versus inpatient admission [17,18].

As Michael Ross notes, “without observation services, many patients are unnecessarily admitted with little benefit from their hospitalization. Others are prematurely discharged with adverse outcomes. In essence, the observation unit has become the 'safety net’ of the emergency department” [19]. Although many hospitals worldwide have had OUs, dedicated ED OUs did not begin until the 1970’s. Chest pain centers, the precursor of today’s OU’s, were established in the early 1980’s [20]. Recognizing that if an OU does not have knowledgeable and accountable leadership implementing a functional operation plan, it will add to rather than resolve ED problems, the American College of Emergency Physicians (ACEP) set up and developed the concept of observation medicine in 1988 [9,21-24]. However, more than two decades later, the quandary of setting up an OU that meets benchmark levels of operation remains an ongoing challenge to hospitals that would benefit from this resource worldwide.

While the concept of a dedicated OU emerged in the United States (US) approximately 30 years ago, it is unclear exactly when this concept was first introduced in Asia. The objective of this study is to define key operational metrics and compare them between three ED OUs in the US and three ED OUs in Asia. We believe that in appreciating the similarities and differences in their operational characteristics, we can formulate ideal targets for these metrics.

## Methods

### Study design and setting

This is a descriptive study of six tertiary care hospitals, all of which are level 1 trauma centers with OUs managed by emergency physicians. The study includes three US hospitals: Brigham and Woman’s Hospital (Boston, MA, US), Beth Israel Deaconess Hospital (Boston, MA, US), and William Beaumont Hospital (Troy, MI, US), and three hospitals in Asia: Queen Mary Hospital (Hong Kong, China), National University Hospital (Singapore), and King Chulalongkorn Memorial Hospital (Bangkok, Thailand). All of these sites are considered leading teaching hospitals in each country and routinely face ED overcrowding.

### Selection of participants

Inclusion criteria were all patients aged 14 and older who were seen in the ED and then subsequently managed in the OU. Data was collected retrospectively from April 2010 to April 2011. This study qualified as a quality improvement initiative and was considered exempt from Institutional Review Board oversight at each institution.

### Outcome measures

Data collected included the hospital’s general descriptive statistics: specifically inpatient hospital beds, acute care ED beds, ED patients/day, and ED patients/month. OU general data included OU beds, OU patients/day, OU patients/bed/day, OU patients/month, location of OU, definition of times for OU length of stay (LOS), maximum allowed OU LOS, OU LOS, OU discharge rate, and OU bed turnover rate. Key operational characteristics included OU policy and protocols, OU staffing model – doctor, nurse, physician assistant, nurse practitioner, and medical assistant work hours, personnel/shift, number of OU beds/personnel, descriptions psychiatric patient care areas, and hours of social worker and case manager availability. We also collected data on the availability of advanced diagnostic tools such as magnetic resonance imaging (MRI) and radiologist interpretations, cardiac stress testing, continuity of care including physical therapy evaluations, and the use of patient satisfaction surveys. Additionally, we collected data on the financial aspects of each OU, including financial incentives for staff via additional payments. Finally, we asked several open-ended questions, such as the strengths and weakness of the OU and other potential alternative uses of OU beds.

### Data collection and processing

We sent a survey with the key data elements described above to the OU medical director of each of the study sites and every site provided data. The OU director was blinded to each of other hospitals’ OU data. The OU director then collected and inserted their hospital’s data and forwarded these results back to the principal investigator. The principal investigator also personally performed site visits to directly observe each OU and conducted interviews with each medical director and other staff members working in the OU, including physicians, advanced practice clinicians, nurses, and medical assistants.

### Primary data analysis

We report the data as percentages and means values. We used SPSS (version 21.0 Somers, NY) to calculate the results with T-tests. The operating characteristics are reported along with 95% confidence intervals (CIs) and around-the-point estimates when relevant.

## Results

We summarize the main differences between key operational characteristics in OUs in the US and Asia in Table 1. Four sites had an OU that is contiguous with the ED while the remaining two OU’s were just a minute walk from the ED.

**Table 1:** Comparison between key operational characteristics of emergency department (ED) observation units (OUs) in the United States and Asia

**Operational characteristic**	**United States**	**Asia**
General data*		
- Hospital beds	809 beds (650–1,026 beds)	1,269 beds (907–1,500 beds)
- ED beds	62 beds (44–86 beds)	Mostly do not have fixed bed
- ED patients/day	421 patients (150–332 patients)	665 patients (120–320 patients)
- OU patients/day	21 patients (17–28 patients)	14 patients (2–24 patients)
- OU beds	17 beds (8–32 beds, some can expand status OU in ED area)	13 beds (8–16 beds)
- Location	Contagious	Contagious and nearby
- How many years since OU started in average (years)	14 years (9–17 years)	3 years (1–6 years)
OU length of stay (LOS), bed turnover rate, and reasons for longer stay		
LOS		
- Definition of times for OU LOS	From the time of the physician order to start observation status (although the patient still in ED) until bed request or discharge status	No clear definition
- Maximum allowed OU LOS	24 hours	24 hours
- Acute care ED LOS prior to OU admission	3–5 hours	2 hours
- OU LOS in average	12.9 hours (13–15 hours)	20.5 hours (15–26 hours, 1 did not answer)
- OU discharge rate	84.3% (83–85%)	88.7% (87–92%)
Bed turnover rate (patients/bed/day) on average	1.6 (0.8–2.1)	0.9 (0.3–1.5)
Reasons why patients stay longer than maximum LOS	Prolonged test results, imaging, consults, limited inpatient psychiatric beds, social work	Completion of patient therapy or clinical improvement, limited inpatient general beds, social work
Characteristic operation in different axes		
Policy and protocol		
- Protocol used in OU	Yes	1 site does not have
- Policy manual available	Yes	1 site does not have
- Policy manual updated annually	1 site does not have	1 site does not have
- Number of OU protocols in use	2, 11, 18	0, 30, 38
- Most common OU protocols	Chest pain, Generic, Abdominal pain	Chest pain, Abdominal pain, Head injury
OU staffing model		
- OU staffing model	MD, PA, NP, RN, MA	MD, RN, MA
- Doctor (MD) availability	Available all the time but physically is 2–4 hours in average	Available all the time but physically is 2–4 hours in average
- Nurse practitioners (NP) and physician assistants (PA)	2 sites have PA, 1 site does not have	Do not have the NP and PA
- Nurses (RN)	Available all the time and 1 RN staffs 5–8 beds	Available all the time and 1 RN staffs 5–8 beds
- Medical assistants (MA)	Yes	1 site has
- The personnel department works in OU, how often?	Work in OU and ED	Work in OU and ED
Psychosocial issues		
- Psychiatric area in ED	1 site has	1 site has
- Psychiatric and suicidal patient accepted in OU area	1 site accepts	1 site accepts
	1 site does not accept	
	1 site does not accept but can be placed in observation status	
- Social worker availability	Daily	Weekdays
- Case manager availability	Daily	2 sites do not have
		1 site has on weekdays
Investigation and continuation of care axis		
- Physical therapy availability	Daily	Weekdays
- Attending emergency radiologist	Daily	2 sites do not have Emergency Radiologist
		1 site has 24/7
- MRI and interpretation	Daily	Only Emergency
- Stress test availability and turn-around time	Yes, 1–3 hours	2 sites do not have
		1 site has, 2 hours
Patient satisfaction		
- OU patient satisfaction measured	Yes	2 sites do not measure
- Instruments used to measure	Press Ganey, Internal questionnaire, phone calls	Questionnaire survey
- Results of OU patient satisfaction survey	1 site 90%	1 site positive but not quantified
	1 site 83%	2 sites no answer
	1 site no answer	
Financial axis		
- Opportunity for additional payment for patients admitted to OU	Yes	No
- Financial incentive for attending physician staff to use OU	Yes	No
- Additional direct payment to attending physician for assigning each patient to OU	1 site has	No
Interrogate and suggestion		
- What happens to OU candidates in your ED if the OU is full	Wait in the ED area	Wait in ED or Direct admission to general ward
- Are OU beds used for other proposes	2 sites use; fast track, inpatient border, procedures	No
	1 site does not use	

*Data reported as mean (Range).

We found that the definitions of OU LOS were different between sites. Within the US, the definition is nearly the same; the observation start time begins from the time an OU bed is requested. In contrast, in Asia there are marked differences between sites and no standard definition for OU start time (one site provided no answer, one site indicated the observation time began when the patient was moved to the OU, and the last site indicated that the observation time began whenever a patient stays in the ED for more than 8 hours). All hospitals allowed for a maximum OU LOS of 24 hours.

We report the main operational metric results in Table 2. OU LOS in the US and Asian sites average 12.9 hours (95% CI, 8.3 to 17.5) and 20.5 hours (95% CI, –49.4 to 90.4) (one site responded “no answer” ), respectively (*P* = 0.39). OU discharge rates in the US and Asian sites average 84.3% (95% CI, 81.5 to 87.2) and 88.7% (95% CI, 81.5 to 95.8), respectively (*P* = 0.11). Bed turnover rates in the US and Asia average 1.56 patients/bed/day (95% CI, -0.1 to 3.3) and 0.9 patient/bed/day (95% CI, -0.6 to 2.4), respectively (*P* = 0.27). None of the differences in OU LOS, OU discharge rate, and bed turnover rate were statistically significant.

**Table 2:** Comparison between key metrics in emergency department (ED) observation units (OUs) in the United States and Asia

	**Mean**	**SD**	**95% CI**	***P *** **value**	**95% CI**	***P *** **value**
**OU patients/bed/day**						
US	1.6	0.7	-0.1 to 3.3	0.58	-0.8 to 2.1	0.27
Asia	0.9	0.6	-0.6 to 2.4	0.12		
**OU length of stay (hours)**						
US	12.9	1.9	8.3 to17.5	0.01	-67.9 to 52.7	0.39
Asia	20.5	7.8	-49.4 to 90.4	0.17		
**OU discharge rate (%)**						
US	84.3	1.2	81.5 to 87.2	0.00	-10.5 to 1.9	0.11
Asia	88.7	2.9	81.5 to 95.8	0.00		

Disease and complaint-specific protocols of care and OU policy manuals are available and updated at least annually in almost every site except one hospital in Asia. The protocols are mostly based on signs and symptoms of disease on ED presentation. The most common OU protocol across all sites is chest pain, while the next top four protocols were abdominal pain/acute gastroenteritis, transient neurological event/vertigo/dizziness, head injury, and generic protocol.

We found various staffing models used across the study sites. An OU staffing model can be composed of various combinations of emergency medicine resident physicians, emergency medicine attending physicians, nurses, medical assistants, nurse practitioners, and physician assistants. In the US, a resident physician, nurse practitioner, or physician assistant manages the OU under attending physician supervision but in Asia the OU is always managed by an attending physician alone. An attending physician was available in all six OUs all the time but physically present in the OU for an average of two to four hours per day. The nurse to bed ratio varied between 5–8 beds/nurse. In general, all OU staff rotate through both the OU and ED as part of their regular work schedule.

Psychiatric or suicidal patients were excluded from management in the physical space of OU from one site in US and one site in Asia. OU social workers and case managers in the US are uniformly available on weekends as well as during the week, but in Asia they are available only on weekdays.

In terms of advanced diagnostic tools and consultant availability, physical therapy, stress testing, real-time attending emergency radiologist over-reads, and advanced imaging such as MRI is routinely available in US OUs (even on weekends), while in Asia these services were not regularly available and when they were available they were limited to weekdays.

Patient satisfaction is uniformly measured in the US, utilizing Press Ganey or other validated survey instruments. In Asia, these data are not routinely captured. Lastly, financial compensation in the form of additional hospital payments and attending physician compensation for OU visits above and beyond the ED evaluation are standard in the US but not available in Asia.

## Discussion

The most notable differences between the ED OUs in the US and Asia were the bed turnover rate (patients/bed/day) and LOS. Both the bed turnover rate and LOS are more favorable in the US compared with OUs in Asia, but these differences were not statistically significant. Prior studies have shown that increasing the availability of diagnostic testing can decrease the observation LOS by facilitating a more rapid diagnosis and thus decreasing the uncertainty and clinical risk when discharging patients [25-28]. Our study similarly demonstrates that more efficient operations may be related to both greater experience – US OUs have been in operation longer, on average since 14 years ago, compared to 3 years in Asia – and greater availability of advanced diagnostics and ancillary and consultant services, especially on weekends. As a result, greater access to these resources may create a more effective OU system in the US than in Asia at this point.

One surprising finding was that the definition of times for OU LOS is different in US and Asia. This may be due to insurance revenue incentives for placing patients in OU which are available only in the US system. The experience of staff in operating an OU and a more prominent role of the emergency physician in the US may also help ensure placement of suitable patients in the OU. This, too, may enhance the rapidity of discharge from the OU.

The number of beds in a dedicated OU should correlate with the annual ED visit volume. Prior data found that OU volume should approximate 4% to 10% of annual ED visits [29]. Therefore, most hospitals in the study still have less OU beds than they should, based on this calculation, but they may be limited by location and the staffing availability.

LOS and bed turnover may also be affected by the OU staffing model, as OU’s in the US are often managed by advanced practice clinicians, such as nurse practitioners or physician assistants with similar patient satisfaction scores [30-39], enabling more rapid turnover due to their continuous physical presence in the OU. This model frees up the responsible attending physician to concentrate on other acute care ED patients, which is a more efficient model than having a dedicated attending physician to attend only to OU patients.

Policy manuals and protocols of care are two of the main operational resources that can also make the OU more efficient [14,40,41]. The ACEP website publishes sample OU protocols from several US hospitals [42]. In our survey, the hospital that did not have a policy manual or protocols of care had an unfavorable bed turnover rate (0.3 patients/bed/day) and LOS (26 hours). Conversely, another hospital having just two protocols (chest pain and general) had a favorable bed turnover rate (2.1 patients/bed/day) and LOS (11 hours). The success of using even just two protocols may be related to data which suggests that chest pain was the most common presentation symptom to the ED patients, representing about 5% to 7% of all visits in the US [43]. Chest pain is the most widely studied OU complaint in the literature and chest pain units have also been the prototype of present day ED OUs since the 1980’s [12,20,44]. Not surprisingly, we found that chest pain was the most common protocol used in all of the OUs, clearly justifying the need for at least a chest pain protocol in every OU.

Prior studies have shown that patient satisfaction is higher in the OU, while the cost is lower when compared to an inpatient unit for similar patients [18,40,41,45-47]. Nevertheless, additional financial incentives for patients managed in an OU varies by country and payer [48]. Our data suggests that paying specifically for OU care may incentivize the hospital and OU to increase efficiency by funding dedicated OU personnel and improving the availability of advanced diagnostics to the OU, which reduces LOS and increases bed turnover [49].

### Limitations

This is a descriptive study and is thus limited in its scope. The small number of study hospitals limits the generalizability of the conclusions of this study. The characteristics of each site may not represent the typical OU for that country. There is also a systematic bias in the data, as all institutions are tertiary care and teaching hospitals, which is not typical of an average hospital and patient population throughout their respective countries. Furthermore, the study collected a snapshot of data at the time of survey administration and patient data was aggregated to means. In addition, OU directors self-reported OU data, which are not publically available and thus we are unable to verify their accuracy. Due to the small number of study hospitals (n = 6), we were unable to find statistical significance between major metrics, such as the OU LOS. This could represent a type II error, as a larger sample size may reveal more clear differences between sites.

## Conclusions

The OU is a resource that can mitigate many of problems of the ED and hospital while simultaneously improving patient care and satisfaction. The key operational characteristics, derived from the experiences, resources, and healthcare systems of the US and Asia, may be a model for the modern ED. These operational characteristics may, in the future, enable hospitals to set up or revise the ED OU and reduce OU LOS, and increase bed turnover and discharge rates while simultaneously improving patient satisfaction and quality of care.

## Abbreviations

ACEP: American College of Emergency Physicians; ED: Emergency department; LOS: Length of stay; MRI: Magnetic resonance imaging; OU: Observation unit; US: United States.

## Competing interests

The authors have no financial or other potential conflicts of interest to disclose.

## Authors’ contributions

AK, BW, and SB contributed to the data collection form. AK collected the data. SG and BW critically revised the manuscript. All authors read and approved the final manuscript.

## Authors’ information

^1^Atthasit Komindr is a director of the emergency medicine observation unit and an attending physician at King Chulalongkorn Memorial Hospital’s emergency unit, Thai Red Cross Society, Bangkok, Thailand. ^2^Christopher W. Baugh is the medical director of Brigham and Women’s Hospital emergency department’s observation unit, assistant professor of emergency medicine at Harvard Medical School, and an attending physician in the Brigham and Women’s Hospital Department of Emergency Medicine, both in Boston, Massachusetts. ^3^Shamai A. Grossman is a vice chair, director of the clinical decision unit, assistant professor of medicine at Harvard Medical School and an attending physician at Beth Israel Deaconess Medical Center Department of Emergency Medicine, Boston, Massachusetts. ^4^ J. Stephen Bohan is executive vice chair of the Brigham and Women’s Hospital Department of Emergency Medicine and director of the department’s Physician Assistant Program. He is also an associate professor at Harvard Medical School and an attending physician at Brigham and Women’s Hospital.
